# Differences in Grain Microstructure and Proteomics of a Broad Bean (*Vicia faba* L.) Landrace Cixidabaican in China Compared with Lingxiyicun Introduced from Japan

**DOI:** 10.3390/plants10071385

**Published:** 2021-07-06

**Authors:** Pengfei Hao, Yaming Zhu, Qidong Feng, Zhuqun Jin, Feibo Wu

**Affiliations:** 1 Department of Agronomy, Institute of Crop Science, College of Agriculture and Biotechnology, Zijingang Campus, Zhejiang University, Hangzhou 310058, China; 11816004@zju.edu.cn (P.H.); 3150100447@zju.edu.cn (Q.F.); wufeibo@zju.edu.cn (F.W.); 2 Cixi Agricultural Technology Extension Center, Cixi 315300, China; eastzym@sohu.com

**Keywords:** phenotype, germplasm resource, iTRAQ, lipoxygenase, electron micrograph, proteomics

## Abstract

In response to the germplasm resources’ conservation in China, the characters of a superior landrace of broad bean (*Vicia faba* L.) Cixidabaican (CX) were identified, compared with Lixiyicun (LX) introduced from Japan. The plant morphology and root structure of CX were larger, pods/seeds number and yield per plant were higher, but the size of pods/seeds and single-seed weight were lower than the similar characteristics in LX. The protein content of dry seeds of CX was 4.1% lower than LX, while the amino acids contents showed no difference between the two cultivars. The seed scan electron micrograph showed that the structure of starch granules was similar, while the granules number was lower in CX than LX. iTRAQ-based proteomics showed that 80 differentially abundant proteins (DAPs) were higher, and 45 DAPs were less abundant in the seeds of CX compared to LX, and DAPs were enriched in proteins of carbohydrate and amino acid metabolism. These results verified the importance of the further study of landraces by showing superior traits of CX, which could contribute to the breeding of better-quality varieties.

## 1. Introduction

Broad bean (*Vicia faba* L.), as one of the most important legume crops, is commonly used as vegetables, non-staple food, and stock feed [1,2]. Several studies also showed that broad bean can improve the fertility of soil through coexistence with specific Rhizobium bacteria, and the legume crop residues can be reused for improving soil conditions by incorporating them as green manure [3,4]. It is also a modern nutritious health food and animal feed, as the seeds are a rich source of proteins, mineral components [5,6], carbohydrates [7], and dietary fiber [8]. The seed protein content ranges from 25% to 30% of dry weight, the second highest among legumes after soybean, and a full suite of essential amino acids can be obtained from broad bean protein, except tryptophan and methionine, while lysine content is highest [9]. The content of starch is also high, 37% to 51.5% of dry weight, but all the nutritional contents vary a lot in different varieties, environments, and cultivation conditions [10]. However, despite the many advantages of broad bean, the global acreage of broad bean has decreased from 3.7 to 2.1 million ha since the 1980s, because of the acceleration of urbanization and the deterioration of environments [11,12].

Germplasm resources, as the landraces of a certain region, have been well-adapted to their local environment, and these resources can be used as donors of various important features in well-designed genetic improvement programs for the creation of new varieties [1]. Thus, local landraces, as important vectors of valuable traits and co-adapted genes, should be well-collected and well-conserved [13]. China, as the largest broad bean producing country, is considered as the secondary center of broad bean genetic diversity, after the Near East [14,15]. Although China possesses abundant germplasm resources of broad bean for future breeding programs, its landraces suffered a lot due to the introduction of excellent new varieties from abroad, and the germplasm resources have been seriously threatened, which has made the collection, evaluation, and conservation of landraces urgent. So far, only a small proportion of broad bean genetic resources have been characterized [16]. Cixidabaican (CX, a landrace of broad bean in Zhejiang province, China) used to be one of the most popular landraces cultivated throughout China, especially in Zhejiang province. CX has been produced for more than 400 years, its whole growth period is about 220 days, the total accumulated temperature need is 2353 ± 81 °C, and plant height can reach about 100 cm at the final flowering stage. The seeds are wide and thin, and suitable for making bean sprouts. However, since the introduction from Japan in 1995 of Lingxiyicun (LX), which has bigger seeds, is softer for fresh eating, and has superior taste, CX has gradually gone out of fashion because of its small seeds and requirements for much more work for planting and cultivation.

To improve the yield and quality of broad bean, large amounts of varietal improvement have been carried out [17]. The collection, identification, and evaluation of germplasm resources, as the material basis of breeding improvement, has aroused great concern to many geneticists and breeders [18]. As the landrace of broad bean in Zhejiang province, China, CX should have lots of well-adapted traits, genes, and proteins for the local environment and management conditions. Consequently, in this paper, the evaluation of CX and LX, including morphology, yield, protein content, amino acids content, and seed characteristics via scanning electron micrography were compared, with the aim to contribute to the conservation of broad bean germplasm resources in China. Proteins are of great importance because of their nutritional and functional properties, where the composition and contents of proteins are not only responsible for taste and nutritional quality, but also for defense against abiotic and biotic stresses. Lampi et al. [19] found that legumes have high activity of lipoxygenase, a key enzyme to start the lipid-degrading LOX pathway, and the main consequences of which are various volatile products, thus influencing the taste of legumes. Zhao et al. reported that broad bean seeds are also rich in natural antioxidant proteins which benefit both humans and animals by defying age and protecting cardiovascular health [20]. In addition, according to Cernay [21], faba bean yield is limited due to biotic and abiotic stresses, while stress resistance is a product of antioxidant protein contents. Thus, we hypothesize that there is a difference in protein expression profiles in CX compared with LX. Therefore, iTRAQ-based proteomics was conducted to identify the protein expression levels in broad bean seeds, which could provide a basis for genetic improvement projects.

## 2. Results and Discussion

### 2.1. Differences in Plant Morphology, Flowers, Stem, Roots, Pods, and Seeds between CX and LX

**Figure 1:**
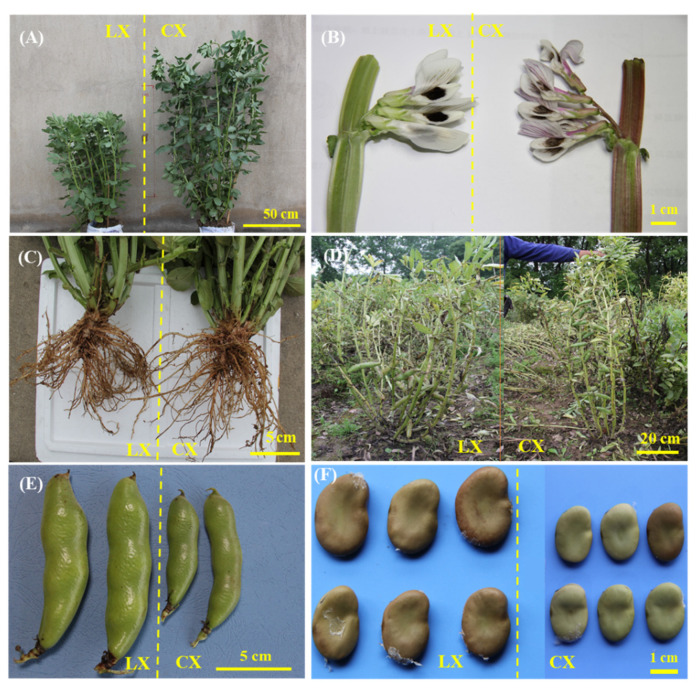
Differences in plant morphology, flowers, stem, roots, pods, and seeds between Lingxiyicun (LX, left panel of each subfigure) and Cixidabaican (CX, right panel of each subfigure). (**A**) Plant morphology at flowering stage, (**B**) flowers and stems, (**C**) roots at flowering stage, and (**D**) whole plant morphology at mature stage. (**E**) Fresh pods and (**F**) dried seeds.

**Figure 2:**
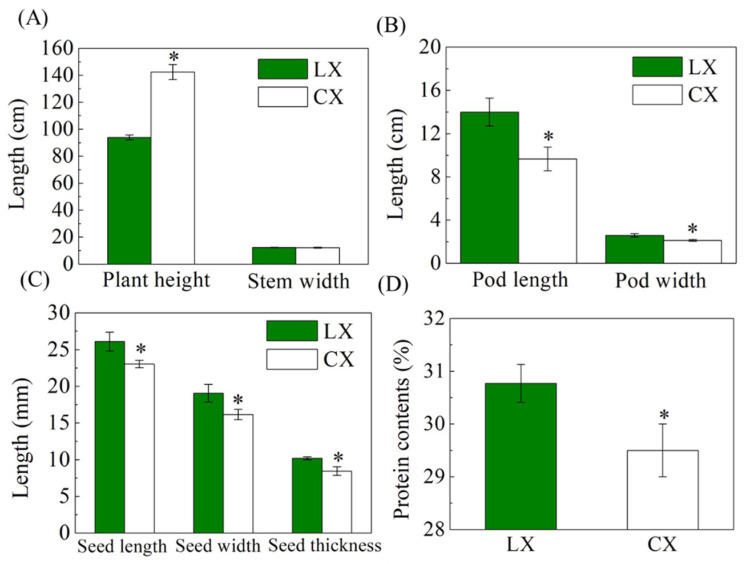
Comparison of (**A**) plant height and stem width, (**B**) pod length and pod width, (**C**) seed length, seed width, and seed thickness at maturity, and (**D**) dry seed protein content between Cixidabaican (CX) and Lingxiyicun (LX). Data are the mean of the two years of data of Year 1: 2018–2019 and Year 2: 2019–2020. * Represents significant difference by Student’s *t*-test at *p* ≤ 0.05. Error bars represent SD value.

According to our results, the development dynamics showed no obvious difference between CX and LX at sowing, emergence, and harvest times, while the time of branching and anthesis of CX occurred 1 or 2 days before LX (Supplementary Table S1). The plant morphologies of CX and LX showed significant differences: on average, of the 2 years of data (i.e., Year 1: 2018–2019 and Year 2: 2019–2020), CX grew 51.7% taller than LX and stem width was smaller at the flowering stage (Figure 1 and Figure 2). The flowers of the varieties showed similar papilionaceous structure and grouping in the inflorescences, although the colors were different. CX flowers were pure white with a black spot in the middle and diffuse anthocyanin among all the petals, and the stems near the petals also showed the presence of anthocyanin in CX, while LX showed no diffuse anthocyanin pigments, which may reflect the fact that CX has relatively higher oxidation and stress resistance [22]. The flowering stage, as the most important development stage of broad bean production, is considered to be the most drought- and cold-sensitive growth stage [23], while the evolution of root structure can readily solve this problem [24]. The root architecture of CX was much larger and longer than LX, which increased the water absorption capacity of CX, and hence, decreased the risk of drought effects (Figure 1C). At the mature stage, the plant phenotype of CX showed a greater plant height, but the fresh pods were significantly smaller than those in LX. The pod length and pod width of CX decreased by 31.1% and 18.2% compared with LX; consequently, the seed size of CX was much smaller than LX. Furthermore, seed pods of CX grew at both the middle and bottom of the plant, while the pods of LX only grew at the bottom, which made the gravitational center of CX higher than LX, and hence increased the risk of lodging (Figure 1D).

### 2.2. Pods, Seeds, and Yield per Plant of CX Were Higher, While the Sizes of Pods and Seeds Were Smaller Than LX

**Figure 3:**
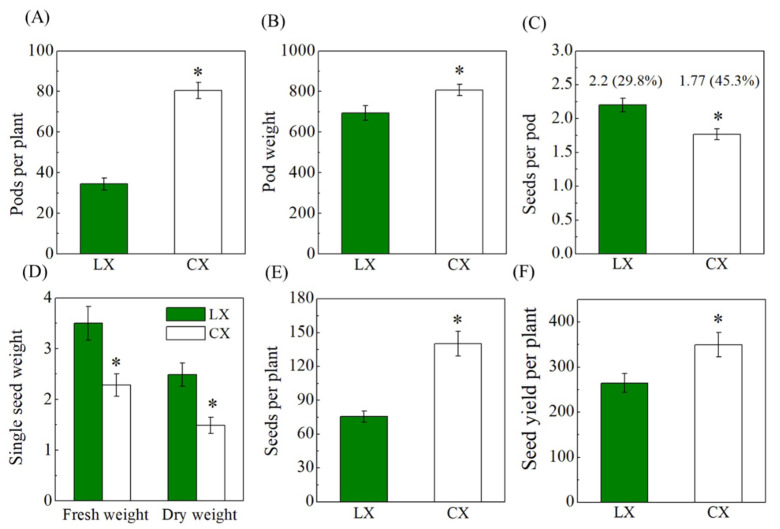
Comparison of yield components. (**A**) Pods per plant, (**B**) pod weight, (**C**) seeds per pod and single seed/pods ratio, (**D**) single-seed fresh and dry weight, (**E**) seeds per plant, and (**F**) seed yield per plant between Cixidabaican (CX) and Lingxiyicun. The percentage value in (**C**) means the single-seed pods ratio. * Represents significant difference between Cixidabaican and Lingxiyicun by Student’s *t*-test at *p* ≤ 0.05.

Yield is the most important characteristic of germplasm resource evaluation. The factors for the evaluation of yield are seed size and weight, pods per plant, and seed number per pod [25]. According to our research, though the plant phenotype of CX was significantly larger than that of LX, the size of the seeds was smaller, and seed length, seed width, and seed thickness were 11.7%, 15.3%, and 17.1% lower compared with LX on average of the two years of data (Figure 2 and Supplementary Table S2). According to the ANOVA shown in Supplementary Tables S2 and S3, the two years’ field tests showed the same trends between the two varieties: CX recorded significantly higher plant height and seeds per plant, with less single-seed dry weight than that of LX, and there was no significant difference in the stem width between the two varieties and the two years. The seeds per pod and single-seed fresh and dry weight were smaller by 19.5%, 28.9%, and 34.6% respectively, while pods per plant, single-seed pod ratio, pod weight per plant, and seed number per plant were significantly higher in CX compared with LX (133.5%, 52.6%, 16.3%, and 85.6% greater, respectively). Altogether, the seed yield per plant was 32.0% higher in CX than LX (Figure 3 and Supplementary Table S3). As to the year effect, the size of pods and seeds, pods and seeds per plant, and seed yield per plant in the Year 2 were higher than those in the Year 1 experiment. Significant interaction effects of year × variety were only found in the traits of pod length, seed length, and seed width. These results indicate that these traits are susceptible to environmental conditions, especially during the seed developing stage while being affected by varieties.

As the result of high plant growth vigor, CX had significantly more pods and greater pod weight per plant, but most of the pods were single-seed pods, with small seeds and lower seed weight, which are not preferred for human consumption. The consensus among the plant breeders and consumers is that larger seeds contain more food reserves and nutritional components [26], hence CX was gradually replaced by LX, in spite of the seed yield per plant of CX being higher than LX. However, according to Etemadi’s report, the currently available varieties showed some drawbacks, such as high seed cost due to the large seed size and relatively low pod yield [27]. Through our research, we have shown that LX had larger seed size, higher single-seed weight, and more seeds per pod, but pods per plant and seeds per plant were lower due to the characteristic of podding only at the bottom, which finally made the yield of LX lower. Even so, several superior traits were demonstrated in CX, such as more plant growth vigor and higher number of pods and seeds per plant, which provided better basics for CX to gain higher yield, and these can be utilized for future breeding to develop larger seed size with higher yield at the same time.

### 2.3. Amino Acid Contents Were High but Showed No Differences between CX and LX

Amino acids are essential nutrients for human beings to maintain normal physiological functions, and both excessive or insufficient intake of essential amino acids will affect human health; hence, the kinds, contents, and proportion of each amino acid are key aspects to evaluate the quality of food. To study the differences of nutritional quality between CX and LX, seed amino acid contents were determined in the Year 1 field experiment (Supplementary Table S4). From the results, methionine and cysteine were the restrictive amino acids, with the contents lower than 100 mg/100 g dry weight. The contents of leucine, lysine, glutamic acid, aspartic acid, and arginine were high, with glutamic acid having the highest content, consistent with the research of Vioque et al. [28]. The proportions of essential amino acids contents vs. total amino acids were 34.7% and 35.5% in CX and LX respectively, which were consistent with the results of Samaei et al. [9] and almost reached the recommended ratio of FAO/WHO/UNU (36%), thus making broad bean a good essential amino acids source for human beings. The amino acids contents of CX were very similar to LX, which showed that CX had as high a nutritional value as the currently popular variety LX.

### 2.4. Seed Crude Protein Contents and Granules Were Lower in CX Than LX

Broad beans have been an essential staple food as an important dietary protein source, widespread in the Mediterranean area, including continental areas such as Syria, modern Iraq, Iran, and in Northwest India, Pakistan, and Southern China. The high contents of digestible proteins and carbohydrates in their seeds mainly explain their extensive food use [29,30]. As an important source of plant protein and carbohydrates for human daily nutrient intake, the seed protein of broad bean is also a valuable aspect for germplasm resource evaluation [31]. According to our research in the Year 1 field experiment, protein content of the dry seeds in CX was significantly lower than LX, by 4.1% (Figure 2).

**Figure 4:**
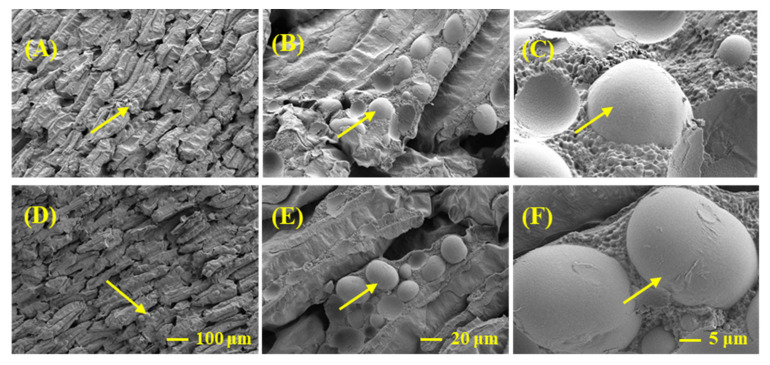
Scanning electron microscopy (SEM) image of mature grains in two broad bean genotypes, Lixiyicun (**A**–**C**) and Cixidabaican (**D**–**F**), at 100- (**A**,**D**), 500- (**B**,**E**), and 2000-fold (**C**,**F**) magnification times, respectively. Arrows represent the starch granules.

The results of the scanning electron microscopy of broad bean cotyledon cross-sections showed that the overall arrangement of tissues was similar in CX and LX, where the shapes of the starch granules were both long elliptical and spheroidal, so we speculated that the granular structure of different faba bean varieties did not contribute to the different taste. However, with regard to starch, the number of granules per unit area was greater in LX than in CX, which might exert a positive influence on superior taste or mouth feel of LX over CX (Figure 4).

### 2.5. Identification of Seed Protein Levels and Differentially Abundant Proteins (DAPs) Significantly Enriched in Carbohydrate and Amino Acid Metabolism

**Figure 5:**
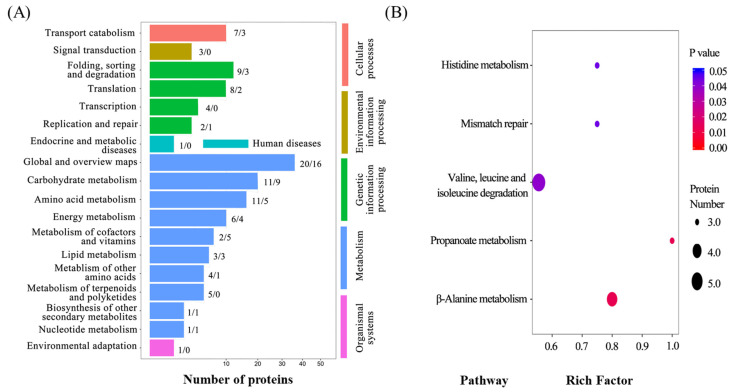
Functional classification (**A**) and pathway enrichment analysis (**B**) of differentially expressed proteins of seeds in Cixidabaican vs. Lingxiyicun. Proteins were identified by iTRAQ-based proteomic analysis, and were classified according to KEGG functional classifications.

The high protein content of broad bean seeds makes these seeds well-adapted to poultry nutrition. Meeting the protein demand of a growing global population is a challenge from both the yield perspective and the quality perspective. While increasing yield potential through breeders’ efforts, it is important to ensure the improvement in protein quantity and quality of broad bean as a protein-rich legume seed [29,30]. Thus, the protein contents and composition are also key factors for plant breeding. Hence, proteomic research was used in the first field experiment to compare the differences in proteins in broad bean seeds between CX and LX, using iTRAQ-based proteomics techniques. A total of 521 proteins were identified, and 125 proteins were differentially abundant proteins (DAPs), among which 80 DAPs were significantly upregulated and 45 DAPs were downregulated in CX compared with LX (Table 1). Thirty up- or down-regulated DAPs remained after the fold change, visualized as heat maps, and the molecular weight (MW), amino acids sequence coverage (AASC), and KOG scores were also recorded (Table 1). To further determine the function of the DAPs, 125 DAPs were classified according to KEGG functional classification, where the top three most enriched functional items were identified as global and overview maps, carbohydrate metabolism, and amino acid metabolism, and the number of up- and down-regulated DAPs are indicated after each item in the color bar (Figure 5). KEGG pathway enrichment results revealed that valine, leucine, and isoleucine degradation, β-alanine metabolism, histidine metabolism, mismatch repair, and propanoate metabolism were significantly enriched DAPs. The interaction of these 5 pathways is shown in Supplementary Figure S1.

Proteins, being the important products of gene expression, are also the reflection of the difference between different germplasm resources [32]. To further identify and evaluate the difference between landrace CX and the excellent imported variety LX, proteomic research of the seeds is urgently needed. As important components of the antioxidant system, the abundance of peroxidase, catalase, and heat shock protein will increase to keep the reactive oxygen species (ROS) in balance when suffering adversity [33]. According to our proteomics results, L-ascorbate peroxidase, 22 kDa heat shock protein, 18.5 kDa heat shock protein, and catalase were 1.53-, 1.52-, 1.45-, and 1.34-fold higher in CX than those in LX, thus ensuring higher stress tolerance, and which may be the reflection of greater environmental adaption ability of the landrace [34].

Enhanced disease susceptibility protein (EDS1) is essential for SA-mediated defenses and is also a major component of SA signaling [35]. The abundance of EDS1 also obtained higher expression in CX, 1.68-fold higher than LX. Thioredoxins are involved a lot in the posttranslational regulation of protein targets in plants, such as seed germination, carbon assimilation, redox signaling, and radical scavenging [36]. The significant high abundance of thioredoxins in CX might increase the ability of seeds in redox modification, hence delaying the senescence and guaranteeing the quality of seeds for a long time.

STICHEL has been reported to play a key role in the regulation of trichome branches in Arabidopsis, which might be directly involved in the formation of branches in the cytoplasm, by mediating the formation of protein complexes, and hence initiating the formation of branches. Trichomes, as the mechanical barrier between plants and the environment, is the first defense of plants to adversity [37]. According to our research, protein STICHEL obtained the highest upregulation in CX compared to LX, which might reflect that CX has high potential to grow more trichome branches than LX, hence increasing the environmental adaptive ability of CX.

In contrast, oleosin protein, histone, and glycinin, which were the important kinds of broad bean proteins, were significantly downregulated in CX compared with LX (Table 1), with the same trends as the seed crude protein contents measured before, which were also decreased in CX compared to LX.

Due to the increasing use of plant proteins from broad beans, a few challenges have appeared, such as the formation of grainy or beany off-flavors [38]. Broad beans are known to have high levels of lipoxygenase (LOX), which acts as the key enzyme of the lipid-degrading LOX pathway [19], with the main results of the LOX pathway being the generation of numerous volatile lipid oxidation products [39]. Great efforts have been undertaken to control the activity of LOX, and hence solve the off-flavor problem [40]. From our results, lipoxygenase in CX was significantly lower than in LX, which might be a good resource for the future study on how to eliminate the beany off-flavors.

## 3. Materials and Methods

### 3.1. Experiment Design, Plant Materials, and Harvest

The field trial was conducted in Cixi, Zhejiang province, China (30.02° N latitude, 121.02° E longitude), with an annual average temperature of 15.9 °C, mean annual precipitation of 1272.8 mm, average annual sunshine of 2038 h, and a subtropical monsoon season. Two broad bean (*Vicia faba* L.) varieties, Cixidabaican (CX, a local landrace commonly grown in Cixi, Zhejiang Province, China) and Lingxiyicun (LX, introduced from Japan in 2017), were used in this experiment, conducted in silty loam soil with uniform and medium fertility. Two-field experiments of 2018–2019 (Year 1) and 2019–2020 (Year 2) respectively, were conducted. Single-seed dibble sowing was used, with 1.0 m row spacing and 0.35 m plant spacing. Five replications were established, with 32 plants per plot (5.6 × 2 m), each containing two rows.

In each plot, 10 plants were randomly selected for pod harvest and yield determination. Pods per plant, seeds per pod and per plant, single-seed pod ratio, pod weight per plant, seed fresh and dry weight, and seed yield were recorded in the mature harvest period.

### 3.2. Determination of Seed Protein and Amino Acid Contents

Seed protein and amino acid contents were measured based on the first field experiment for the following ultrastructure observation and proteomics analysis. Dry and healthy seeds were selected for protein content measurement. After removing the seed coat carefully, the seeds were placed in a whirlwind pulverizer FG-11 and ground into powder, and then sealed tightly for future use. A total of 1 g of broad bean powder was weighed for crude protein content measurement, according to the Kjeldahl method (AOAC, 2006). A total of 0.2 g of powder was weighed for amino acid content determination using an AAA-400 amino acid analyzer according to Lisiewska [8]. The proportion of essential amino acids to total amino acids was determined as follows:

E/T% = (IlE + Leu + Lys + Met + Phe + Thr + Val + Try)/(IlE + Leu + Lys + Met + Phe + Thr + Val + Try + Ala + Arg + Asp + Cys + Glu + Gly + His + Pro + Ser + Tyr) × 100%.(1)

### 3.3. Ultrastructure Observation of Broad Bean Cotyledon Cross-Sections

The ultrastructure of broad bean cotyledon cross-sections was observed through scanning electron microscopy according to Chen et al. [41], with minor modifications. In brief, the seed coat was carefully removed, and the cotyledons were cut to obtain a slice with natural fracture surfaces remaining. The fracture surfaces were sputter-coated with gold and palladium, and the ultrastructure of the natural fracture surface of each variety was observed using a GeminiSEM 300 (Oberkochen, Germany). To compare the differences in ultrastructure between CX and LX, 100-, 500-, and 2000-fold magnifications were used, especially for the structure and arrangement of starch granules.

### 3.4. Proteomics Analysis

The fresh broad bean seeds of CX and LX were harvested and sampled at the mature stage in the Year 2018–2019, and a total of 3 replicates were used. A subsample of 0.8 g of seeds was taken for the preparation of protein, and for the quantification, concentration detection and digestion, iTRAQ-labeling and peptide fractionation, HPLC and mass spectrometer detection were conducted according to Zeng et al. [42]. The proteins were identified via NCBI (https://www.ncbi.nlm.nih.gov/ (accessed on 29 May 2019)), where the taxonomy search was for *Vicia faba*, on 28 May 2019. The raw MS/MS data were searched using Mascot version 2.3.02 against the selected database, and the variable modifications of amino acids were oxidation and iTRAQ8plex and the fixed modifications were carbamidomethyl and iTRAQ8plex. The overview of protein identification is shown in Supplementary Table S5. Bioinformatics analysis of the proteomic data was as described by Zeng et al. [42]. The quantitative ratios (CX/LX) of the identified proteins greater than 1.2 were considered as upregulated (*p* ≤ 0.05), while quantitative ratios less than 0.83 were considered as downregulated (*p* ≤ 0.05). The mass spectrometry proteomics data have been deposited to the ProteomeXchange Consortium (http://proteomecentral.proteomexchange.org (accessed on 29 May 2019)) via the iProX partner repository, with the dataset identifier PXD026415 [43].

### 3.5. Statistical Analysis

The data were presented as mean values of each treatment. Statistical analysis was performed using Data Processing System (DPS) statistical software. Student’s *t*-tests were performed to evaluate significant variety differences between CX and LX at the significance level of *p* ≤ 0.05 [44]. Origin version 7.5 was used for the preparation of graphs.

## 4. Conclusions

Increasing urbanization and environmental change has led to decreases in cultivation and the disappearance of several conventional local genetic resources, thus making it imperative that germplasm resources are conserved as a source for plant breeding [45]. China, as the country with the largest cultivation of broad beans and the second center of broad bean genetic resources, has lots of excellent landraces and traits for breeders to use to improve currently popular varieties; hence, there is an urgent need to evaluate the landraces of China. In this paper, the morphology, yield, protein contents, amino acid contents, starch contents, and proteomes of landrace Cixidabaican (CX) were identified and compared with Lingxiyicun (LX), which was introduced from Japan. In summary:

(1) CX grew taller than LX, but the seed size of CX was smaller than LX, and seed weight was lower.
(2) Seed yield of CX was higher due to a greater number of pods, but the protein content was lower than LX.
(3) Proteomics analysis showed that, as a result of long-term adaption to the local environment, heat shock proteins, L-ascorbate peroxidase, catalase, EDS1 protein, thioredoxins, and STICHEL protein were upregulated, which could indicate increased environmental adaption ability.
(4) Downregulated LOX activity in CX can also be a useful trait for future work on the alleviation of off-flavors.

In conclusion, although CX has been replaced by LX nowadays, this landrace still possesses many excellent traits, which may be important for future breeding improvements. For example, because of the long-term adaption to the local environment, environmental responsive proteins were upregulated in CX, and these proteins and related genes can be utilized for breeding development through genetic engineering to endow the varieties introduced from foreign areas with a higher ability for environmental adaption. Cultivation techniques, such as hybridization, backcross techniques, and population design in open pollinated conditions, are also expected to increase genetic gains, and hence combine beneficial characteristics (e.g., higher yield and lower off-flavors) of CX with LX, and consequently, develop tasteful varieties with higher yield.

## Figures and Tables

**Table 1:** Differentially abundant proteins in seeds of Cixidabaican (CX) compared with those in Lingxiyicun (LX).

Protein_ID	Fold Change	Description	MW (Da)	AASC (%)	Score
XP_006581893.1	3.46	Protein STICHEL-like 2	109,238.8	1.1	652
XP_025981111.1	2.55	Probable ATP synthase 24 kDa subunit, mitochondrial	20,383.14	6.2	n
XP_003523436.1	2.46	Aldehyde dehydrogenase family 3 member H1	53,299.19	3.3	695
NP_001236192.2	2.38	Profilin-2	14,167	9.9	223
XP_003537765.3	2	50S ribosomal protein L12, chloroplastic	19,744.65	4.3	195
XP_006577714.1	1.88	Peroxiredoxin-2E-1, chloroplastic-like	22,543.65	5.3	204
XP_025984179.1	1.81	Brefeldin A-inhibited guanine nucleotide-exchange protein 2	158,613.8	0.6	2165
XP_003517606.1	1.81	Pre-sequence protease 2, chloroplastic/mitochondrial	122,069.5	1.1	1672
XP_003525659.1	1.75	Nucleoside diphosphate kinase 2, chloroplastic	25,211.09	4.0	296
YP_538747.1	1.73	Ribulose-1,5-bisphosphate carboxylase/oxygenase large subunit	53,014.61	4.6	n
XP_003529252.1	1.69	Probable N-acetyl-gamma-glutamyl-phosphate reductase	43,088.54	2.8	513
XP_003527042.1	1.68	Protein EDS1L	70,738.43	1.0	n
NP_001341836.1	1.62	2-cys peroxiredoxin	28,437.57	2.3	398
XP_006575386.1	1.59	60S ribosomal protein L10	25,486.29	2.7	409
XP_014629812.1	1.56	Eukaryotic translation initiation factor 5A3 isoform X1	14,929.39	5.9	240
NP_001336714.1	1.54	40S ribosomal protein S20-2	13,738.35	9.8	224
NP_001235587.1	1.53	L-ascorbate peroxidase 2	27,147.82	3.6	n
XP_014619949.1	1.52	Putative bZIP domain class transcription factor isoform X1	53,525.58	2.0	n
NP_001235130.2	1.52	Heat shock 22 kDa protein, mitochondrial isoform 2	23,722.07	5.2	230
XP_003539349.1	1.49	CBS domain-containing protein CBSX1, chloroplastic	25,106.35	4.4	50.1
XP_014634736.1	1.45	Probable aldo-keto reductase 2	37,788.32	4.1	564
NP_001343409.1	1.45	Putative 18.5 kDa class I heat shock protein	17,364.88	21.1	201
XP_003537679.1	1.43	Phosphoserine aminotransferase 1, chloroplastic	45,349.77	2.7	664
XP_003525794.1	1.42	Hsp70-Hsp90 organizing protein 1-like	63,722.89	3.4	841
NP_001237987.2	1.37	Aspartate aminotransferase isoform X1	50,703.81	2.8	778
NP_001341770.1	1.36	Putative triosephosphate isomerase	27,391.21	5.1	429
XP_006599411.1	1.35	V-type proton ATPase subunit C	42,660.65	2.4	642
XP_003548332.1	1.35	Thioredoxin H9 isoform X2	15,563.91	8.0	199
NP_001341767.1	1.35	Phosphoglycerate kinase	42,347.63	14.5	723
NP_001240021.1	1.34	Catalase	57,024.44	13.4	926
XP_003550821.1	0.81	Leghemoglobin reductase-like	53,178.63	2.6	860
XP_003525164.1	0.8	Ribonuclease TUDOR 1-like	108,891.7	1.0	1453
NP_001237229.1	0.79	40S ribosomal protein S13	17,168.51	11.3	280
XP_003540396.1	0.79	Ketol-acid reductoisomerase, chloroplastic	63,668.43	4.3	n
NP_001235189.1	0.79	Lipoxygenase	96,336.78	3.0	n
XP_003528976.1	0.79	Dihydropyrimidine dehydrogenase (NADP (+))	46,554.31	10.8	706
XP_003517743.1	0.79	Polygalacturonase 1 beta-like protein 3	68,652.63	2.2	n
NP_001235936.2	0.78	Superoxide dismutase	15,322.56	19.7	254
XP_003531110.3	0.78	1-Cys peroxiredoxin	24,523.66	4.6	325
NP_001241357.1	0.78	Phosphoenolpyruvate carboxylase isoform X1	111,129.3	1.6	n
XP_003520940.1	0.78	Frataxin, mitochondrial	21,874.16	5.8	169
XP_003531426.1	0.77	Thioredoxin-like protein Clot	15,204.9	6.1	166
XP_003526464.3	0.77	Oleosin 1	17,516.01	3.6	n
XP_003542149.1	0.77	Mitochondrial import inner membrane translocase subunit Tim13	9538.618	11.6	87
XP_003543938.1	0.76	α-1,4 glucan phosphorylase L isozyme	110,522.1	7.9	1528
XP_003543443.1	0.76	Probable histone H2B.3	14,656.14	8.3	216
XP_003526742.1	0.75	Probable 6-phosphogluconolactonase 4, chloroplastic	27,821.5	3.5	342
XP_003519681.1	0.75	Mitochondrial import inner membrane translocase subunit TIM10	9895.614	20.2	134
XP_003554323.1	0.73	Hypersensitive-induced response protein 2-like isoform X1	31,797.14	9.8	506
XP_003555839.1	0.72	Probable prefoldin subunit 5	16,900.77	7.7	223
XP_003538225.2	0.71	Subtilisin inhibitor CLSI-I	13,362.84	6.7	n
NP_001237169.1	0.71	Seed maturation protein PM41	8211.974	20.5	n
XP_003522597.1	0.7	DNA mismatch repair protein MLH1	82,357.54	4.8	1066
XP_006600683.1	0.7	Delta-1-pyrroline-5-carboxylate dehydrogenase 12A1	50,380.97	4.7	762
XP_003529967.1	0.68	Ubiquitin carboxyl-terminal hydrolase 6 isoform X2	54,583.49	1.7	730
XP_003521095.1	0.68	α-L-arabinofuranosidase 1	74,340.09	1.9	n
NP_001238008.1	0.68	Glycinin G4 precursor	64,196.62	3.0	n
XP_014625942.1	0.68	Histone H4, partial	11,068.2	22	163
NP_001347984.1	0.65	Glyceraldehyde-3-phosphate dehydrogenase	36,914.17	16.6	618
NP_001340170.1	0.6	Alcohol dehydrogenase family protein	41,619.82	7.1	677

The fold change was the ratio obtained by comparing the relative protein abundance in CX with LX (CX vs. LX). Fold change ≥ 1.2 are more abundant, between 0.83 < fold change < 1.2 are unchanged, and fold change ≤ 0.83 are less abundant, *p*-value ≤ 0.05. n represents null. MW, molecular weight; AASC, amino acid sequence coverage; Score, KOG score.

## Data Availability

Not applicable.

## Supplementary Materials

The following are available online at https://www.mdpi.com/article/10.3390/plants10071385/s1, Table S1: Development dynamics of Cixidabaican (CX) and Lingxiyicun (LX) during the whole growth period. Table S2: Amino acid composition of Cixidabaican (CX) and Lingxiyicun (LX), in mg/100 g seed dry weight. Figure S1: Pathway interactions among Valine, leucine, and isoleucine degradation, β-Alanine metabolism, Histidine metabolism, Mismatch repair, and Propanoate metabolism. Table S3: Comparison of pods number per plant, single-seed pods ratio, pod weight per plant, seeds number per pod, single seed fresh weight, single seed dry weight, seeds number per plant and seed yield per plant between Cixidabaican (CX) and Lingxiyicun (LX), and the ANOVA analysis of the effect of year, variety and their interactions. Table S4: Amino acid composition of Cixidabaican (CX) and Lingxiyicun (LX), in mg/100 g seed dry weight. Table S5: Overview of protein identification by iTRAQ.
