# Supplementary material for: Differences in Grain Microstructure and Proteomics of a Broad Bean (Vicia faba L.) Landrace Cixidabaican in China Compared with Lingxiyicun Introduced from Japan
Source: Plants (Basel). 2021 Jul 6;10(7):1385. doi: 10.3390/plants10071385 (PMC8309391; doi:10.3390/plants10071385)
Supplement: Supplementary file 1 [file plants-10-01385-s001.zip › Supplementary materials R4.pdf]

Table S1 Development dynamics of Cixidabaican (CX) and Lingxiyicun (LX) during the whole growth period.

Variety	Sowing	Emergence	Branch	Anthesis	Harvest
Year 1: 2018- 2019					
LX	Oct 24th	Nov 10th	Jan 19th	March 2nd	Apr 27th, May 3rd
CX	Oct 24th	Nov 8th	Jan 18th	Feb 28th	Apr 27th, May 3rd
Year 2: 2019-2020					
LX	Oct 25th	Nov 12th	Jan 20th	March 4th	Apr 29th, May 3rd
CX	Oct 25th	Nov 9th	Jan 19th	March 2nd	Apr 29th, May 3rd

Table S2 Comparison of plant height, stem width, pod length, pod width, seed length, seed width and seed thickness between Cixidabaican (CX) and Lingxiyicun (LX), and the ANOVA analysis of the effect of year, variety and their interactions.

Variety	Plant height (cm)	Stem width (mm)	Pod length (cm)	Pod width (cm)	Seed length (mm)	Seed width (mm)	Seed thickness (mm)
Year 1: 2018- 2019							
LX	94.33	12.50	14.42	2.48	25.96	18.87	9.91
CX	143.00 ***	12.20	9.58 ***	1.99 ***	22.44 ***	15.32 ***	8.07 ***
Year 2: 2019-2020							
LX	93.41	12.29	13.57	2.67	26.19	19.27	10.49
CX	141.73 ***	12.07	9.71 ***	2.22 ***	23.63 ***	16.97 ***	8.83 ***
Average of the 2 Years Field Experiments							
LX	93.87	12.39	14.00	2.58	26.08	19.07	10.19
CX	142.37 ***	12.14	9.65 ***	2.11 ***	23.04 ***	16.15 ***	8.45 ***
	**F value / P**						
Year	0.49 / ns	4.56/ns	12.62/*	36.45/**	16.81/**	99.78/**	32.57/**
Variety	955.53 / ***	7.47/ns	1620.05/***	208.18/***	325.36/***	872.17/***	224.46/***
Year × Variety	0.01/ns	0.07/ns	20.06/**	0.38/ns	8.17/*	42.44/***	0.35/ns

*, **, and ***, Significance at $P \leq 0.05$, 0.01 and 0.001, respectively; ns, no significance.

Table S3 Comparison of pods number per plant, single-seed pods ratio, pod weight per plant, seeds number per pod, single seed fresh weight, single seed dry weight, seeds number per plant and seed yield per plant between Cixidabaican (CX) and Lingxiyicun (LX), and the ANOVA analysis of the effect of year, variety and their interactions.

Variety	Pods per plant	Pod weight per plant (g)	Seeds per pod	Single seed fresh weight (g)	Single seed dry weight (g)	Seeds per plant	Seed yield per plant (g)
Year 1: 2018- 2019							
LX	34.21	727.90	2.08	3.37	2.24	70.8	237.8
CX	79.52 ***	856.51 ***	1.68 ***	2.43 ***	1.45 ***	133.8 ***	325.6 **
Year 2: 2019-2020							
LX	34.67	659.87	2.32	3.62	2.31	80.32	291.64
CX	81.33 ***	757.47 ***	1.86 ***	2.54 ***	1.53 ***	146.67 ***	373.39 **
Average of the 2 Years Field Experiments							
LX	34.44	639.89	2.20	3.50	2.28	75.56	264.72
CX	80.43	807.00	1.77 ***	2.49 ***	1.49 ***	140.24 ***	349.50 **
	**F value/P**						
Year	0.248/ns	24.53/**	8.75/*	12.76/*	4.82/ns	5.22/ns	8.02/*
Variety	295.26/***	45.04/***	36.695/***	378.91/***	484.37/***	176.12/***	22.22/**
Year × variety	0.06/ns	0.82/ns	0.22/ns	1.91/ns	0.002/ns	0.10/ns	0.03/ns

*, **, and ***, Significance at $P \leq 0.05$, 0.01 and 0.001, respectively; ns, no significance.

Table S4 Amino acid composition of Cixidabaican (CX) and Lingxiyicun (LX), in mg/100 g seed dry weight.

Amino acid	Cixidabaican (CX)	Lingxiyicun (LX)
Valine (Val)	486.97 ± 30.25	500.83 ± 50.10
Tyrosine (Tyr)	215.19 ± 15.76	218.13 ± 37.38
Threonine (Thr)	401.83 ± 13.84	385.11 ± 28.31
Serine (Ser)	487.93 ± 14.32	463.20 ± 29.39
Proline (Pro)	346.42 ± 12.66	356.30 ± 30.90
Phenylalanine (Phe)	445.67 ± 19.25	454.94 ± 43.02
Methionine (Met)	43.01 ± 9.18	36.26 ± 15.13
Lysine (Lys)	740.18 ± 28.15	741.66 ± 66.36
Leucine (Leu)	778.15 ± 32.56	759.20 ± 64.24
Isoleucine (Ile)	413.17 ± 28.20	426.58 ± 42.84
Histidine (His)	287.58 ± 11.11	284.80 ± 27.33
Glycine (Gly)	458.36 ± 19.18	449.89 ± 36.54
Glutamic (Glu)	1800.32 ± 70.43	1721.10 ± 126.99
Cystine (Cys)	66.01 ± 6.33	71.49 ± 5.67
Aspartic (Asp)	1108.46 ± 51.74	1029.71 ± 82.38
Arginine (Arg)	976.84 ± 41.24	945.70 ± 96.23
Alanine (Ala)	472.69 ± 16.26	469.20 ± 38.08
E/T (%)	0.347 ± 0.002	0.355 ± 0.003

E/T (%) represent the proportion of essential amino acids to total amino acids.

Table S5 Overview of protein identification by iTRAQ.

Sample	Total spectra	Spectra	Unique spectra	Peptide	Unique peptide	Protein
Vicia faba	844,748	2,013	1,919	809	775	521

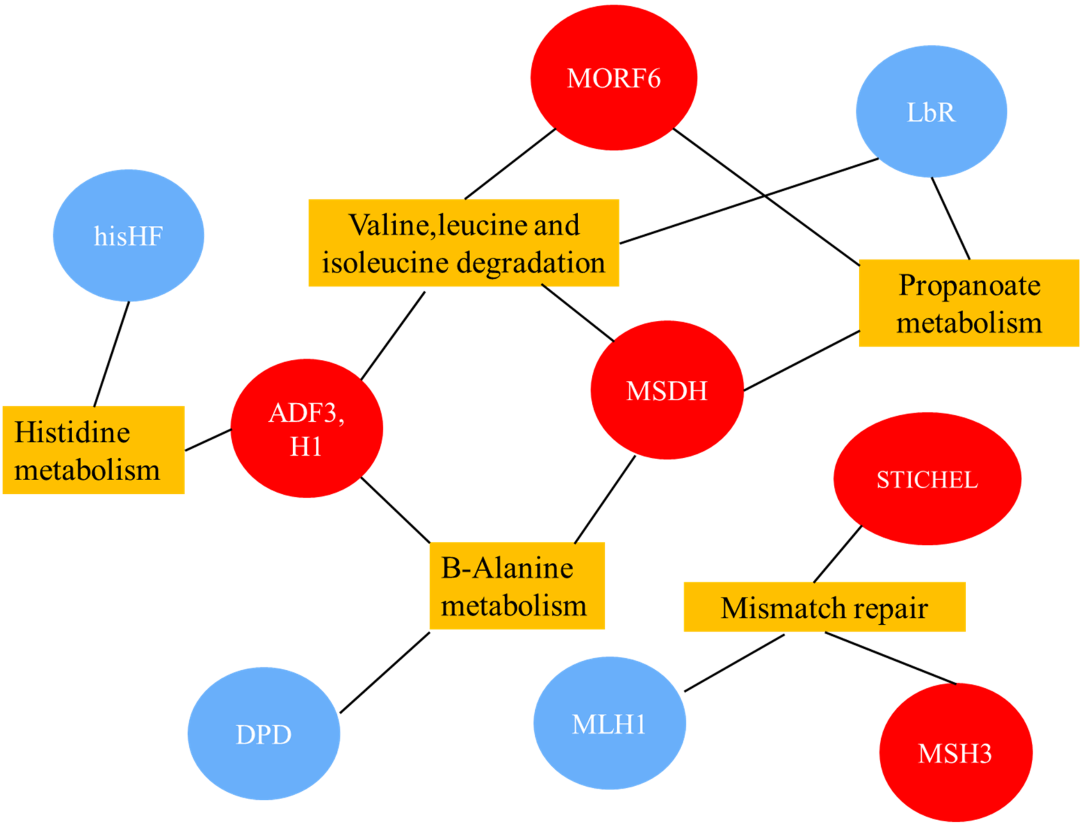

Figure S1 Pathway interactions among Valine, leucine and isoleucine degradation, β-Alanine metabolism, Histidine metabolism, Mismatch repair and Propanoate metabolism. Orange squares represent pathways. Proteins in red and blue circle represent significant higher- and lower-expression. hisHF, imidazole glycerol phosphate synthase hisHF; MOF6, multiple organellar RNA editing factor 6-like; LbR, leghemoglobin reductase-like; ADF3, aldehyde dehydrogenase family 3 member H1; MSDH, methylmalonate-semialdehyde dehydrogenase [acylating]; STICHEL, protein STICHEL-like 2; DPD, dihydropyrimidine dehydrogenase (NADP (+)); MLH1, DNA mismatch repair protein MLH1; MSH3, DNA mismatch repair protein MSH3 isoform X2.
