# Supplementary figures and images for: Differences in Grain Microstructure and Proteomics of a Broad Bean (Vicia faba L.) Landrace Cixidabaican in China Compared with Lingxiyicun Introduced from Japan
Source: Plants (Basel). 2021 Jul 6;10(7):1385. doi: 10.3390/plants10071385 (PMC8309391; doi:10.3390/plants10071385)

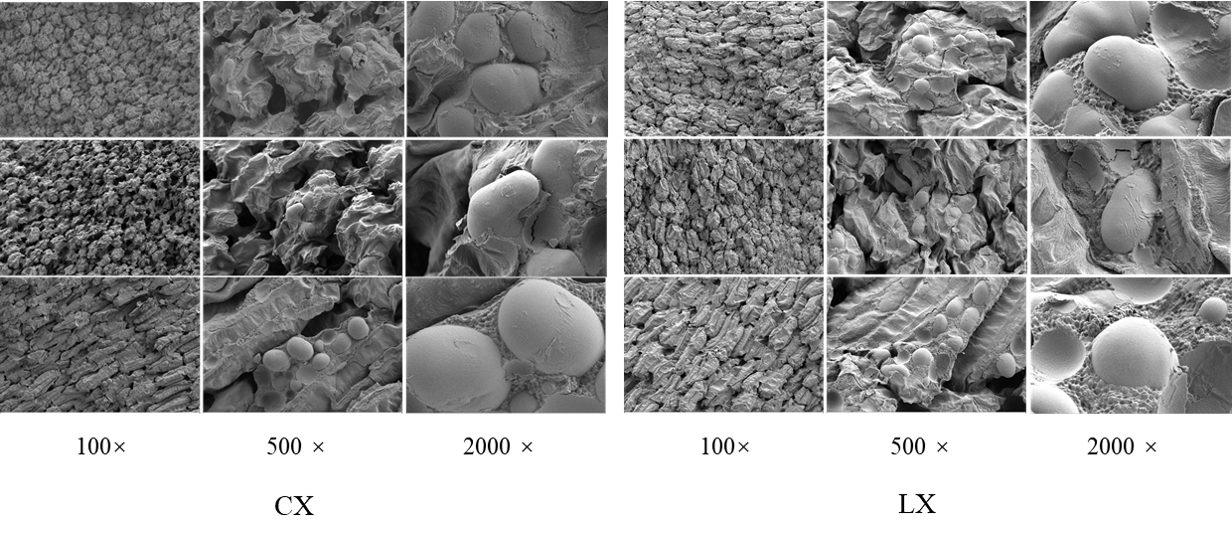

Supplement: Supplementary file 1 [file plants-10-01385-s001.zip › scanning electron microscopy.png]
